# Verbal Autopsy: Evaluation of Methods to Certify Causes of Death in Uganda

**DOI:** 10.1371/journal.pone.0128801

**Published:** 2015-06-18

**Authors:** Arthur Mpimbaza, Scott Filler, Agaba Katureebe, Linda Quick, Daniel Chandramohan, Sarah G. Staedke

**Affiliations:** 1 Child Health & Development Centre, College of Health Sciences, Makerere University, Kampala, Uganda; 2 Infectious Diseases Research Collaboration, Kampala, Uganda; 3 Global Fund to Fight AIDS, Tuberculosis and Malaria, Geneva, Switzerland; 4 Centers for Disease Control and Prevention, Atlanta, Georgia, United States of America; 5 London School of Hygiene & Tropical Medicine, London, United Kingdom; Tulane University School of Public Health and Tropical Medicine, UNITED STATES

## Abstract

To assess different methods for determining cause of death from verbal autopsy (VA) questionnaire data, the intra-rater reliability of Physician-Certified Verbal Autopsy (PCVA) and the accuracy of PCVA, expert-derived (non-hierarchical) and data-driven (hierarchal) algorithms were assessed for determining common causes of death in Ugandan children. A verbal autopsy validation study was conducted from 2008-2009 in three different sites in Uganda. The dataset included 104 neonatal deaths (0-27 days) and 615 childhood deaths (1-59 months) with the cause(s) of death classified by PCVA and physician review of hospital medical records (the ‘reference standard’). Of the original 719 questionnaires, 141 (20%) were selected for a second review by the same physicians; the repeat cause(s) of death were compared to the original,and agreement assessed using the Kappa statistic.Physician reviewers’ refined non-hierarchical algorithms for common causes of death from existing expert algorithms, from which, hierarchal algorithms were developed. The accuracy of PCVA, non-hierarchical, and hierarchical algorithms for determining cause(s) of death from all 719 VA questionnaires was determined using the reference standard. Overall, intra-rater repeatability was high (83% agreement, Kappa 0.79 [95% CI 0.76-0.82]). PCVA performed well, with high specificity for determining cause of neonatal (>67%), and childhood (>83%) deaths, resulting in fairly accurate cause-specific mortality fraction (CSMF) estimates. For most causes of death in children, non-hierarchical algorithms had higher sensitivity, but correspondingly lower specificity, than PCVA and hierarchical algorithms, resulting in inaccurate CSMF estimates. Hierarchical algorithms were specific for most causes of death, and CSMF estimates were comparable to the reference standard and PCVA. Inter-rater reliability of PCVA was high, and overall PCVA performed well. Hierarchical algorithms performed better than non-hierarchical algorithms due to higher specificity and more accurate CSMF estimates. Use of PCVA to determine cause of death from VA questionnaire data is reasonable while automated data-driven algorithms are improved.

## Introduction

Verbal autopsy (VA) is an indirect method of determining cause of death based on an interview with the caretakers of a deceased individual, which has been widely used to collect information on cause-specific mortality where vital registration systems are lacking and medical information on deaths is incomplete [1]. Different approaches of determining cause of death from VA interview information exist, including physician review, algorithms, and more recently, computerized coding of VA (CCVA) which can either be algorithmic or probabilistic in approach [2–4]. However, the optimal approach for determining causes of death from VA data is unclear, and has been the subject of debate [1,3,5].

The most widely used method is physician review, known as physician-certified VA (PCVA), in which physicians are trained to review questionnaire data and determine cause of death. Although the validity of PCVA has been evaluated [6], concerns about the repeatability of PCVA have been raised [4,7]. The level of agreement on causes of death certified by two independent physicians from VA (inter-rater repeatability) has been extensively studied [5,8–15]. However, very few published studies have assessed the repeatability of causes of death certified from VA by the same physicians at different time points (intra-rater reliability) [16].

An alternative to PCVA for determining cause(s) of death from VA data are algorithms. Algorithms can be expert-derived or data-driven. Expert algorithms include a set of pre-defined diagnostic criteria developed by a panel of physicians,based on experience or review of existing literature [2]. Alternatively, data-driven algorithms are derived from existing data using standard statistical techniques including logistic regression, decision tree algorithms, and bayesian classification, which identify discriminatory functions of indicators to be included in an algorithm [2]. Algorithms can be used to guide physicians as they review VA questionnaires and classify cause(s) of death; alternatively, algorithms may be computerized to automate the process [3,17]. Several algorithms based on expert opinion or derived from data have been developed, but their accuracy has been shown to vary widely, and may be lower than that of PCVA [18–22]. In addition to algorithms, probabilistic approaches have been developed [3]. Unlike algorithmic approaches that assess the presence or absence of single cause of death based on positive or negative responses to symptom-related questions, automated methods apply probabilistic reasoning adjusting the probability of a range of multiple possible outcomes simultaneously [2]. Like algorithms, probabilistic methods can be expert driven or data driven [23]. Recent reports suggest that automated probabilistic approaches outperformor are equivalent to PCVA [24, 25], but these results have been disputed [23,26].

Data from a VA validation study conducted in three epidemiological settings in Uganda were used to investigate the performance of different methods for determining causes of death from VA data. We evaluated the intra-rater reliability of PCVA, and also compared the accuracy of PCVA to that of two algorithms; one developed with the input of expert physicians (non-hierarchical) and another data-driven (hierarchical).

## Materials and Methods

The VA data-set used to investigate the performance of different methods for determining causes of death was obtained from a VA validation study that was approved by the Ugandan National Council for Science and Technology, the Centers for Disease Control and Prevention, and the ethics committees of Makerere University Faculty of Medicine, and the London School of Hygiene and Tropical Medicine. Details of the VA validation study method are published elsewhere [27]. Briefly, the study was conducted from 2008–2009 in selected public hospitals located in three districts; Tororo (high malaria transmission) Kampala (medium transmission) and Kisoro (low transmission). Deaths among hospitalized children aged less than five years, including neonatal deaths were registered over a period of one year and VA interviews were conducted with appropriate caretaker of children. PCVA was used for determining cause of death following World Health Organization (WHO) standards at the time [28]. The reference standard for assessing the accuracy of PCVA was the cause of death determined by physician review of hospital medical records at each site. The sensitivity, specificity and positive predictive value, and accuracy of cause specific mortality fraction (CSMF) estimates of the PCVA method for determining cause of death were computed for a select group of common causes of childhood death for each site. Analysis and presentation of results was stratified by two age groups: 1) Neonatal deaths (0–28 days), and 2) Childhood deaths (1–59 months)

### Intra-rater reliability of PCVA

Twenty percent of VA questionnaires were systematically sampled for assessment of intra-rater reliability. Using a list of sequentially ordered identification numbers for each site, we systematically selected every fifth VA questionnaires with the corresponding COD originally determined by physician review of the data. VA questionnaires were re-evaluated by the original physician a second time. Re-determination of causes of death from VA questionnaires occurred 3–9 months after the original assessment, and physicians were blinded to the causes of death recorded in the original VA death certificate.

### Development of non-hierarchical algorithms

The non-hierarchical algorithms were based on previously published expert algorithms [19,21,29–31]. Seven physicians who reviewed the original VA questionnaires were asked to review existing algorithms and develop a refined algorithm (including the criteria for diagnosis) taking into account diagnostic criteria that they used to attribute malaria and other common childhood illness as cause of death when originally reviewing VA questionnaires. The non-hierarchical algorithms underwent a final round of review by a team of the investigators, including a pediatrician and three epidemiologists. Each algorithm consisting of a pre-determined set of diagnostic criteria to be applied to VA questionnaire data; specific combinations of the presence or absence of certain signs and symptoms experienced prior to death indicating different causes of death. For neonatal causes of death, non-hierarchical algorithms were developed for the following causes of death: 1) septicemia, 2) meningitis, 3) pneumonia, and 4) congenital malformation. Final non-hierarchical algorithms for childhood deaths were limited to the most common causes of death, including 1) malaria, 2) pneumonia, 3) meningitis, 4) diarrheal illnesses, 5) malnutrition, and HIV/AIDS (Table 1).

**Table 1:** Algorithms used for determining cause(s) of death from verbal autopsy questionnaires.

Cause of death	Algorithm criteria
**CHILDREN AGED > 27 DAYS**	
Malaria	Fever +
	—Convulsions without stiff neck OR bulging anterior fontanelle OR
	—Unconscious without stiff neck OR bulging anterior fontanelle Or
	Fever +
	—Convulsions without loss of consciousness OR
	—Difficulty breathing OR
	—Blood in urine OR
	—Pale body OR
	—Lack of blood
HIV/AIDS	—Mouth sore OR
	—Yellow discoloration of eyes OR
	—Wasting
Pneumonia	Cough for less than 22 days +
	—Fever AND
	—Difficulty breathing
Meningitis	Stiff neck OR
	Bulging anterior fontanelle +
	—Fever OR
	—Convulsion OR
	—Unconscious
Diarrheal illnesses	More than 2 loose, watery stools
Malnutrition	Wasted OR
	Weight loss (for ≥ 14 days) OR
	Swelling +
	—Rash OR
	—Change in hair color
**CHILDREN AGED < 28 DAYS (NEONATES)**	
Septicemia	Any two of the following:
	— Stopped suckling
	— Fever or cold to touch
	— Unresponsive or unconscious or lethargic
	— Convulsions
	— Vomiting
	— Skin bumps containing blisters or single large area of pus
Meningitis	Fever and convulsions
Pneumonia	Cough + Difficulty in breathing
Congenital malformations	Any specified deformity

### Development of hierarchical algorithms

**Fig 1:**
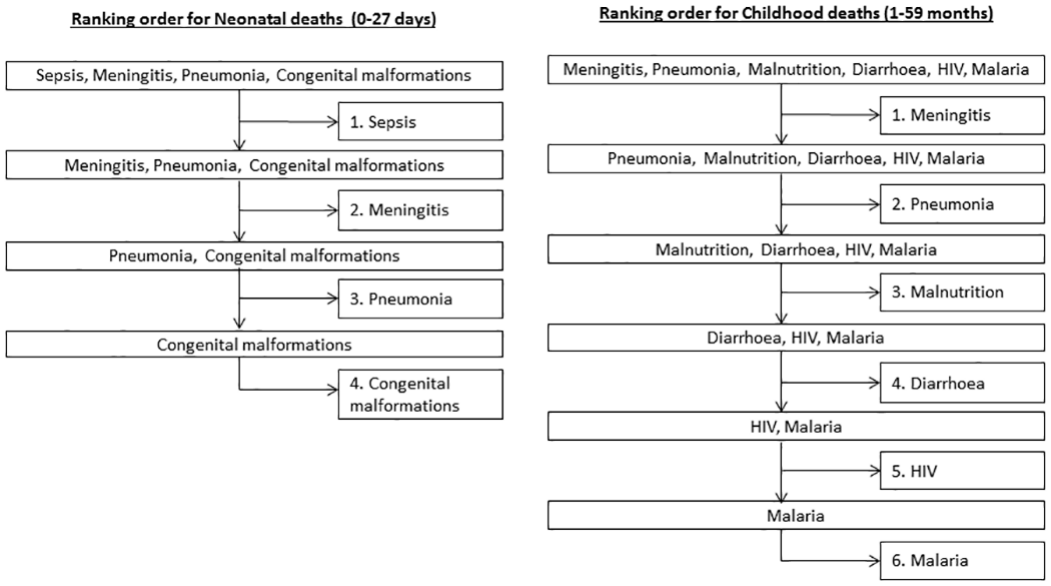
Ranking order for Hierarchical algorithms for childhood and neonatal deaths.

Hierarchical algorithms were developed by ranking the performance of the non-hierarchical algorithms to reach common causes of childhood deaths, including neonatal deaths. Ranking was prioritized based on specificity of causes of death as determined using expert algorithms. The cause of death with the highest specificity wasplaced at the top of the hierarchy while the least specific was placed at the bottom (Fig 1). Neonatal deaths were ranked in the following order: (1) septicemia, (2) meningitis, (3) pneumonia, and (4) congenital malformations. Childhood causes of death were ranked as follows: (1) meningitis, (2) pneumonia, (3) malnutrition, (4) diarrhea, (5) HIV, and (6) malaria.

### Data analysis

#### Intra-rater reliability of PCVA

The cause of death determined by physicians upon repeat review of VA questionnaires was compared to the cause of death originally determined by the same physician. The percentage level of agreement and Kappa statistic was calculated using Stata 12 (StataCorp, College Station, Texas, USA) for each physician. Interpretation of Kappa values was based according to the criteria of Landis and Kock [32], who recommended that a Kappa value greater than 0.8 be considered ‘almost perfect’, between 0.6 and 0.8 ‘substantial’, between 0.4 and 0.6 ‘moderate’, between 0.2 and 0.4 ‘fair’, between 0 and 0.2 ‘slight’, and between 0 and -1 ‘poor.’ Furthermore, to assess the impact of re-determination of cause of death on the CSMF attributable to malaria and other common illness at the population level we compared the CSMF (CSMF _Original_) to the re-determined CSMF (CSMF _Repeat_).

### Validation of algorithms

A database comprised of responses to closed-ended sections of VA questionnaires, and the reference causes of death derived from medical records were generated. Causes of death determined by non-hierarchal algorithms were derived by applying non-hierarchal algorithms to the closed-ended sections of VA questionnaires. Non-hierarchal algorithms were capable of classifying more than one cause of death. Hierarchal algorithms were also applied to the same VA questionnaire database, generating a single cause of death for each questionnaire.

The sensitivity and specificity of each method for determining cause of death were calculated by comparing the cause of death assigned by each method to the ‘reference standard’ for causes of death derived from hospital medical records, including malaria, pneumonia, diarrhea, meningitis, malnutrition, and HIV. CSMF estimates of the leading causes of death were also calculated for PCVA (CSMF_PCVA_), non- hierarchical algorithms (CSMF_NHA_) and hierarchal algorithms (CSMF_HA_). The difference between the CSMF determined using each of the three methods and the ‘reference standard’ (CSMF_MR_) was calculated for the common causes of death. For neonatal and childhood deaths, where algorithms were developed for five and four commonest causes of death respectively, causes of death that did not fit the commonest cause of death list were categorized as ‘others’ and were factored in all analysis.

## Results

### Intra-rater reliability of PCVA

**Fig 2:**
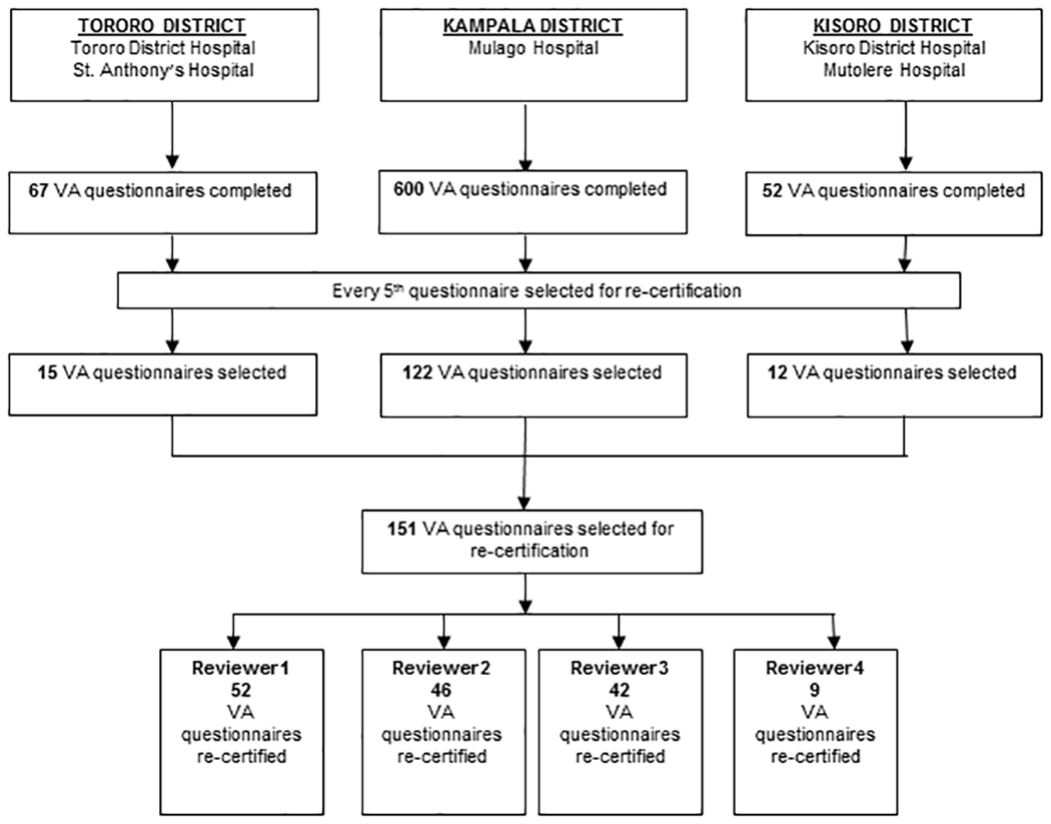
Trial profile: selection of VA questionnaires for re-assigning cause of death by physician reviewers.

A total of 149 VA questionnaires were selected for re-determining cause of death by four physician reviewers, each with a different number of VA questionnaires (Fig 2). Although the performance of individual physicians varied, intra-rater reliability was almost perfect for physician reviewer ‘2’ (Kappa statistic = 0.87) and substantial for physician reviewer ‘1’ and ‘3’ (Kappa statistic = 0.77, respectively) and moderate for physician reviewer ‘4’ (Kappa statistic = 0.52). Overall, the level of agreement was substantial (Kappa statistic = 0.79) (Table 2). The repeat estimates of CSMF for the different causes of death did not differ substantially (< 10%) when compared to the original CSMF estimated by the same reviewer (Table 3).

**Table 2:** Physician reviewer intra-rater reliability coefficients.

		Kappa		
Physician reviewer	Percentage agreement (%)	Kappa statistic	95%CI	p-value
**All**	83%	0.79	0.76–0.82	< 0.001
**1**	81%	0.77	0.77–0.84	< 0.001
**2**	89%	0.87	0.82–0.95	< 0.001
**3**	81%	0.77	0.68–0.96	< 0.001
**4**	67%	0.52	0.13–0.65	0.004

**Table 3:** Level of agreement in CSMF upon repeat determination of cause of death by physician reviewers.

Diagnosis	Cause Specific Mortality Fraction (CSMF)		
	CSMF_ORIGINAL_	CSMF_REPEAT_	(CSMF_REPEAT_−CSMF_ORIGINAL_)
**All**			
**Malaria**	13%	11%	-2%
**Pneumonia**	8%	10%	+2%
**Meningitis**	15%	15%	0
**Diarrhea**	9%	9%	0
**Malnutrition**	19%	12%	-7%
**Reviewer 1**			
**Malaria**	17%	14%	-3%
**Pneumonia**	5%	14%	+9%
**Meningitis**	12%	14%	+2%
**Diarrhea**	0	0	0
**Malnutrition**	29%	14%	-15%
**Reviewer 2**			
**Malaria**	13%	11%	-2%
**Pneumonia**	6.5%	4%	-2.5%
**Meningitis**	17%	17%	0
**Diarrhea**	22%	22%	0
**Malnutrition**	13%	13%	0
**Reviewer 3**			
**Malaria**	6%	6%	0
**Pneumonia**	12%	12%	0
**Meningitis**	19%	17%	-2%
**Diarrhea**	6%	6%	0
**Malnutrition**	19%	11.5%	-7.5%
**Reviewer 4**			
**Malaria**	44%	33%	-11%
**Pneumonia**	11%	11%	0
**Meningitis**	0	0	0
**Diarrhea**	0	0	0
**Malnutrition**	11%	0	-11%

### Accuracy of PCVA, non-hierarchical algorithms and hierarchal algorithms for neonatal deaths

**Fig 3:**
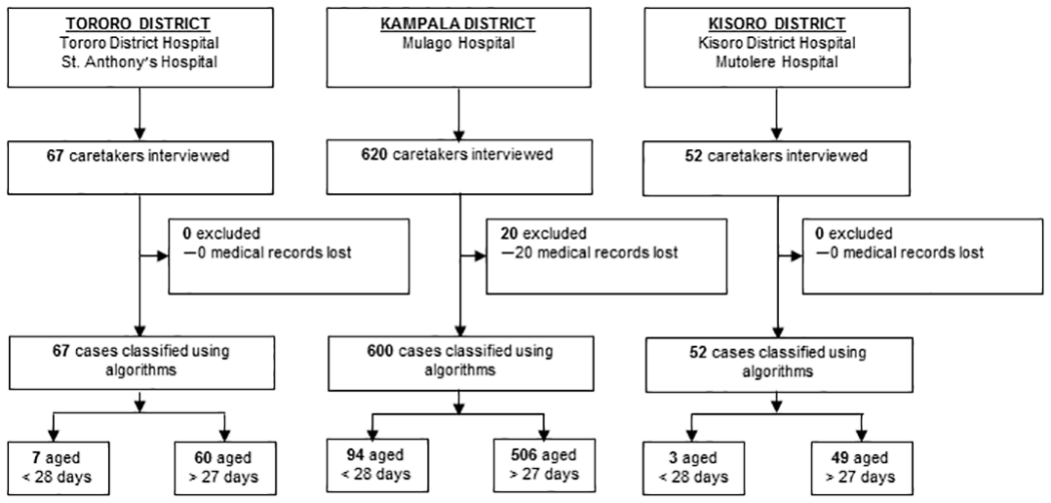
Trial profile: selection of VA questionnaires to be assigned cause of death using algorithms.

A total of 104 questionnaires representing neonatal deaths were evaluated using algorithms (Fig 3). Based on PCVA, common causes of death among neonates included septicemia (29%), meningitis (38%), pneumonia (8%), and congenital malformations (6%). Sensitivity of PCVA, non-hierarchical algorithms, and hierarchical were generally low (<50%) for the four major causes of neonatal deaths, with exception of the sensitivity of non-hierarchical algorithms (76%) for septicemia deaths, and PCVA (61%) for meningitis deaths. For congenital malformation, pneumonia, and septicemia deaths, specificity of PCVA was high (97%, 93%, and 78% respectively), and comparable to that of hierarchical algorithms (94%, 88%, and 52% respectively). With the exception meningitis deaths where the specificity score of non-hierarchical algorithms (79%) was high, for the other causes of neonatal deaths the specificity of non-hierarchical algorithms (<20%) was very low (Table 4).

**Table 4:** Sensitivity and specificity of different methods of determining cause of death from VA questionnaires.

	PCVA		Non-hierarchical algorithms		Hierarchical algorithms	
	Sensitivity	Specificity	Sensitivity	Specificity	Sensitivity	Specificity
**CHILDHOOD DEATHS (1–59 months)**						
**ALL SITES**						
Malaria	61%	88%	84%	34%	16%	93%
Pneumonia	42%	92%	35%	75%	30%	80%
Meningitis	48%	94%	47%	84%	47%	84%
Diarrhea	35%	94%	76%	59%	30%	87%
Malnutrition	56%	89%	64%	69%	33%	81%
HIV/AIDS	61%	95%	70%	47%	2%	92%
**TORORO**						
Malaria	62%	84%	82%	39%	14%	94%
Pneumonia	33%	94%	0	78%	0	80%
Meningitis	75%	95%	50%	84%	50%	84%
Diarrhea	0	95%	0	44%	0%	76%
Malnutrition	53%	91%	87%	82%	27%	91%
HIV/AIDS	0	92%		47%	0	95%
**KAMPALA**						
Malaria	60%	88%	84%	34%	18%	93%
Pneumonia	41%	91%	38%	75%	33%	80%
Meningitis	47%	94%	49%	84%	49%	84%
Diarrhea	31%	94%	74%	60%	27%	88%
Malnutrition	58%	89%	59%	68%	32%	80%
HIV/AIDS	61%	96%	70%	47%	2%	92%
**KISORO**						
Malaria	0	88%	0	35%	0	96%
Pneumonia	55%	93%	30%	75%	25%	83%
Meningitis	43%	95%	29%	85%	29%	86%
Diarrhea	75%	95%	100%	66%	63%	93%
Malnutrition	40%	86%	80%	64%	60%	77%
HIV/AIDS	0	0	0	49%	0	94%
**NEONATAL DEATHS (0–28 days)**						
**ALL SITES**						
Septicemia	44%	78%	76%	15%	47%	52%
Meningitis	61%	68%	48%	79%	35%	87%
Pneumonia	9%	93%	12%	9%	9%	88%
Congenital malformation	50%	97%	12%	17%	17%	94%

CSMF estimates for congenital malformation and pneumonia deaths were accurate and comparable for PCVA (0%, and -3% difference respectively), non-hierarchical algorithms (1%, and 2% difference respectively), and hierarchical algorithms (1% and 2% difference respectively). Non-hierarchical algorithms (50% difference), and hierarchical algorithms (16% difference) overestimated the CSMF for septicemia deaths compared to PCVA (-3% difference) that performed best. On the contrary non-hierarchical algorithms (5% difference), and hierarchical algorithms (-4% difference) had better CSMF estimates for meningitis deaths compared to PCVA (-16% difference, Table 5).

**Table 5:** CSMF and level of agreement of different methods of determining cause of death from VA questionnaires.

	ReferenceStandard (CSMF_MR_)	Cause Specific Mortality Fraction			% level of agreement		
		PCVA (CSMF_PCVA_)	Non-hierarchical algorithms(CSMF_NHA_)	Hierarchal algorithm (CSMF_HA_)	CSMF_PCVA_−CSMF_MR_	CSMF_NHA_−CSMF_MR_	CSMF_HA_−CSMF_MR_
**CHILDHOOD DEATHS (1–59 months)**							
**ALL SITES**							
Malaria	12%	18%	68%	12%	6%	56%	0
Pneumonia	21%	15%	26%	22%	-6%	5%	1%
Meningitis	10%	10%	19%	19%	0	9%	9%
Diarrhea	13%	9%	46%	15%	-4%	33%	2%
Malnutrition	16%	19%	36%	21%	3%	20%	5%
HIV/AIDS	7%	8%	54%	7%	1%	47%	0
**TORORO**							
Malaria	48%	38%	71%	15%	-10%	23%	-33%
Pneumonia	20%	15%	20%	18%	-5%	0	-2%
Meningitis	7%	10%	18%	18%	3%	11%	11%
Diarrhea	2%	5%	55%	16%	3%	53%	14%
Malnutrition	25%	20%	35%	13%	-5%	10%	-12%
HIV/AIDS	0	8%	53%	5%	-8%	53%	5%
**KAMPALA**							
Malaria	10%	17%	68%	12%	7%	58%	2%
Pneumonia	10%	8%	28%	22%	-2%	18%	12%
Meningitis	10%	10%	19%	19%	0	9%	9%
Diarrhea	14%	9%	45%	16%	-5%	31%	2%
Malnutrition	16%	19%	36%	22%	3%	20%	6%
HIV/AIDS	9%	9%	54%	8%	0	45%	-1%
**KISORO**							
Malaria	0	12%	65%	8%	12%	65%	8%
Pneumonia	40%	27%	26%	20%	-13%	-14%	-20%
Meningitis	14%	10%	16%	16%	-4%	2%	2%
Diarrhea	16%	16%	45%	16%	0	29%	0
Malnutrition	10%	16%	41%	27%	6%	31%	17%
HIV/AIDS	0	0	49%	6%	0	49%	6%
**NEONATAL DEATHS (0–27 days)**							
**ALL SITES**							
Septicemia	32%	29%	82%	48%	-3%	50%	16%
Meningitis	22%	38%	27%	18%	16%	5%	-4%
Pneumonia	10%	7%	12%	12%	-3%	2%	2%
Congenital malformation	6%	6%	7%	7%	0%	1%	1%

### Accuracy of PCVA, non-hierarchical algorithms and hierarchal algorithms for causes of childhood deaths

A total of 615 questionnaires representing childhood deaths were evaluated using algorithms (Fig 3). The accuracy of PCVA, non-hierarchical algorithms and hierarchical algorithms ranged widely depending on the cause of death and the site (Table 4). For malaria deaths, the sensitivity of non-hierarchical algorithms (84%) was higher than that of PCVA (61%) and hierarchical algorithms (16%). This pattern was consistent in Kampala and Tororo. In contrast, the specificity of non-hierarchical algorithms for determining malaria deaths was low in Kampala (34%) and Tororo (39%), and much lower than the specificity of PCVA (84–88%) and hierarchal algorithms (93–94%) in determining malaria deaths (Table 4). Sensitivity and specificity of all methods for determining diarrheal deaths followed a pattern similar to that observed in determining malaria deaths. Sensitivity and specificity of non-hierarchical algorithms in determining pneumonia and meningitis deaths were comparable to hierarchal algorithms but lower when compared to PCVA at all sites (Table 4).

CSMF estimates of non-hierarchical algorithms (CSMF_NHA_) deviated greatly from the reference standard (CSMF_MR;_ difference > 10%), with a tendency to overestimate the CSMF for the leading causes of death across all sites. The CSMF estimated by PCVA (CSMF_PCVA_) and the hierarchal algorithms (CSMF_HA_) approximated that of the reference standard (CSMF_MR_) for all cause(s) of death, performing far better than non-hierarchical algorithms. However, overall CSMF estimates of malaria deaths were best approximated by hierarchal algorithms (0% difference), exceeding performance of both PCVA (6% difference) and non-hierarchical algorithms (56% difference), which both overestimated the fraction of deaths attributable to malaria when compared to the reference standard (Table 5). This pattern was consistent across all sites with the exception of Tororo, where PCVA was more accurate.

## Discussion

To investigate the performance of different methods for determining causes of death from previously collected VA data, we evaluated the intra-rater reliability of PCVA, and compared the accuracy of PCVA and two algorithms, using physician review of hospital medical records as a reference standard. Contrary to prior reports, our findings suggest that the intra-rater reliability for classifying cause of death using PCVA is high [7,33]. Reliability of 3 out of 4 physicians was classified as ‘substantial’, and repeat CSMF estimates for common causes of death were similar to the original estimates. One physician’s score was sub-optimal possibly due to low number of records reviewed by the physician. Regardless, the overall performance was good with a Kappa score indicating ‘substantial’ agreement between reviews. The physicians’ prior knowledge of local epidemiology likely contributed to the good performance by three physicians [2]. Although prior knowledge and subjective application of clinical judgment may be considered as ‘biases’, they are likely to have had a positive impact on the physicians’ ability to correctly identify cause of death [34]. However, the subjectivity of the PCVA method may limit the ability to apply temporal and spatial comparisons of mortality data. Standardized training of physician reviewers addresses this concern to an extent [11].

Although use of algorithms has been advocated to overcome the issue of subjectivity, the accuracy of algorithms remains a concern [4]. For neonatal deaths, sensitivity of PCVA, non-hierarchical algorithms, and hierarchical algorithms was low (<50%) for all the causes of neonatal deaths, with exception of meningitis with PCVA (61%). On the contrary, specificity of PCVA and hierarchical algorithms performed well compared to non- hierarchical algorithms, although specificity was relatively low for meningitis with PCVA (68%) and for septicemia with hierarchical algorithms (52%). In terms of estimating CSMF, all three methods were relatively accurate with exception of non-hierarchical algorithms and hierarchical algorithms which overestimated the CSMF for septicemia deaths, a fact probably attributed to the low specificity of non-hierarchical algorithms and hierarchical algorithms in determining septicemia deaths.

For childhood deaths, compared to PCVA, sensitivity of non-hierarchical algorithms was impressive, particularly for classification of malaria, diarrheal and malnutrition deaths. However, sensitivity was gained at the expense of specificity. This imbalance between sensitivity and specificity undermined the performance of the non-hierarchical algorithms when estimating CSMF for common causes of death resulting in gross overestimation of the CSMF for respective causes of death. Importantly, we note that the degree of error in estimating the CSMF was inversely proportional to the specificity level attained, implying that error in estimating CSMF reduced as specificity increased. With exception of septicemia deaths, this phenomenon was not observed with neonatal deaths. Overlap of signs and symptoms of common illnesses used to develop diagnostic criteria for these diseases could have limited the ability of the algorithms to distinguish between illnesses resulting in assignment of multiple cause(s) of death and a marked decline in specificity.

Hierarchical algorithms assigning a single cause of death from each VA questionnaire resulted in an increase in specificity of the algorithm in determining causes of death, but at the expense of sensitivity which declined. However, compared to the non- hierarchal algorithms, hierarchal algorithm estimates of the reference CSMF were accurate and as good as those of PVCA for all the common causes of death; a fact attributed to the high specificity levels of hierarchal algorithms. This finding, previously described by Anker et al [35], demonstrated that specificity is an important driver of the accuracy of CSMF estimates determined by these methods. However, superiority was apparent only when the reference CSMF level was low (~ < 10%) for a particular disease [35]. In Tororo and Kisoro, the reference CSMF levels for malaria and pneumonia deaths were very high and hierarchal algorithms, despite low specificity, greatly underestimated the CSMF attributable to malaria and pneumonia deaths at these sites suggesting that benefits of increased specificity in estimating the CSMF are only applicable when the true CSMF is low. Indeed, this may explain why non-hierarchal algorithms and hierarchal algorithms overestimated septicemia deaths among neonates. The primary limitation of either algorithm is their inflexibility. Unlike physicians, algorithms lack ‘clinical acumen’ and are not capable of interpreting the potential contribution of multiple disease processes ultimately leading to death. This limitation of algorithms is well-recognized, and has been cited as the primary disadvantage of algorithms and other automated methods for determining cause(s) of death from VA data [4].

Several computerized methods premised on different algorithmic methods (expert driven, data driven; Tariff, Artificial Neural Network, and Random Forest), probabilistic (expert driven; InterVA, Data drive; King-Lu, and Simplified Symptom Pattern) approaches have been developed as alternative methods of determining cause(s) of death from VA questionnaires [23,28,30,33,36–38]. The dataset used to validate the Tariff, Random Forest, King-Lu and Simplified Symptom Pattern methods was comprised of a randomly selected number of gold standard hospital deaths that formed part of a larger multi-country verbal autopsy validation study [39]. In these validation studies, all three methods were more accurate than PCVA for most of the causes of death [36,37,40]. However these results have been disputed, with a systemic review of 19 studies finding that no single VA method outperformed the other across selected CODs for both individual and population-level COD assignment [23].

InterVA uses a probability matrix, which was derived from clinical knowledge of group of physicians [41], and in addition to the TARIFF method, has been recommended by the World Health Organization in their 2012 VA guidelines as one of preferred methods for determining cause(s) of death [42]. However, two studies validating the performance of InterVA compared to PCVA against a gold standard based on rigorously defined clinical criteria yielded conflicting results; one study conducted in Kilifi on the coast of Kenya showed that InterVA performed as well as PCVA in determining the top five underlying causes of death in a rural community, the other study based on a multisite validation study showed that InterVA performance was suboptimal compared to PCVA [5,43]. Although InterVA has been widely implemented [44–47], inconsistent reports of the performance of this method, as well as alternative CCVA approaches, should not be overlooked. Until CCVA methods are improved and evaluated, consistently yielding more accurate results than PCVA, it is likely that PCVA will continue to be used widely to determine causes of death from verbal autopsy questionnaires [23].

Our study is not without limitation. Internal evaluation of the performance of the hierarchical algorithm may have biased results, showing good performance of the hierarchical algorithms. However, the results of our analysis are strengthened by the inclusion of three different study sites. Furthermore, the small sample of deaths among some of the causes of the death in both neonates and children, especially when stratified by site, may have undermined our ability to detect representative estimates of measures of performance.

## Conclusions

Our study provides insights into the performance of different methods for determining cause(s) of death from VA questionnaire data collected in three sites. Importantly, we demonstrate that repeatability of PCVA is high, contrary to expectation, and that overall PCVA performed well. Thus, based on our results and available evidence so far, PCVA remains a reliable method for determining cause of death from VA questionnaire data. Given the lack of consensus on the accuracy of recently developed CCVA methods, PCVA still has a place in determining cause of death in VA, while existing and newer automated data-driven algorithms, which undoubtedly would be more efficient, are further developed, refined, and evaluated.
